# Secretion of Fc-amidated peptide fusion proteins by Chinese hamster ovary cells

**DOI:** 10.1186/s12896-015-0173-5

**Published:** 2015-06-27

**Authors:** Kristina R. Carlson, Steven C. Pomerantz, Jiali Li, Omid Vafa, Michael Naso, William Strohl, Richard E. Mains, Betty A. Eipper

**Affiliations:** Department of Neuroscience, UCONN Health Center, 263 Farmington Avenue, Farmington, CT 06030-3401 USA; Biologics Research, Biotechnology Center of Excellence, Janssen Research & Development, LLC, Spring House, PA 19477 USA; Biologics Research, Janssen Research & Development, San Diego, CA 92121 USA; Department of Molecular Biology and Biophysics, UCONN Health Center, Farmington, CT 06030 USA

**Keywords:** Amidation, CHO cells, Mass spectrometry, Glucagon-like peptide 1, Peptide YY, Neuromedin U

## Abstract

**Background:**

The therapeutic use of α-amidated peptides (e.g. calcitonin, glucagon-like peptide) has increased dramatically, but there are major impediments to wider use of such peptides. Larger peptides are expensive to synthesize, and short plasma half-lives frequently limit the clinical circumstances in which the peptides would be useful. Both problems are potentially solved by producing peptides as fusions with the Fc region of human immunoglobulin.

**Methods:**

Glucagon-like peptide 1 (GLP1), peptide YY (PYY) and neuromedin U (NMU) were expressed and purified from stable CHO lines; since the α-amide group is essential for full biological potency of many peptides, Fc-fusion peptides were expressed in CHO lines stably expressing the α-amidating enzyme, peptidylglycine α-amidating monooxygenase (PAM: EC 1.14.17.3). Purified fusion proteins were analyzed intact and after HRV3C rhinovirus protease cleavage, at a site in the linker separating the Fc region from the peptide, by mass spectrometry and amide-specific immunoassays.

**Results:**

The Fc fusions were expressed at 1–2.5 μg/mg cell protein and secreted at 5-20 % of cell content per hour, in a peptide-specific manner. CHO cells express measurable endogenous PAM activity, amidating 25 % of Fc-PYY and almost 90 % of Fc-GLP1. Expression of exogenous PAM increased the level of peptide amidation to 50 % of Fc-PYY and 95 % of Fc-NMU. The Fc-GLP1 fusions were 10,000-fold less active than synthetic GLP1 in a cell-receptor cyclic AMP-based assay, as expected since the amino terminal of GLP1 is essential for full biological activity. The Fc-PYY fusions were 100-fold less active than PYY-NH_2_ but 10-fold more active than non-amidated PYY-Gly.

**Conclusions:**

This type of approach can be used for the production of stabilized α-amidated peptides aimed at clinical trials.

## Background

The peptides used for intercellular communication are generally short-lived, with half-lives of only a few minutes. The constant region of human immunoglobulin (Fc, fragment crystallizable) is long-lived in the circulation (2 weeks or more) and has been used to prolong the half-lives of many proteins [1]. Many bioactive peptides terminate with an essential C-terminal α-amide [2, 3]. C-terminal α-amidation requires the α-hydroxylation of a peptidylglycine precursor by peptidylglycine α-hydroxylating monooxygenase (PHM) and subsequent cleavage of the α-hydroxylated intermediate by peptidyl-α-hydroxyglycine α-amidating lyase (PAL) to produce the amidated product and glyoxylate. A bifunctional type 1 integral membrane protein, peptidylglycine α-amidating monooxygenase (PAM), is the only enzyme known to catalyze this reaction [3, 4].

We wanted to determine whether Fc-amidated peptide fusion proteins could be produced from Fc-peptidylglycine precursors and secreted efficiently and whether the products would be biologically active. Chinese hamster ovary (CHO) cells are widely used for the production of recombinant antibodies and up to 70 % of all biopharmaceuticals [5]. CHO cells were also used to produce PHM and PAL for crystallographic studies [6, 7]. The CHO cell genome encodes PAM splice variants similar to those found in rat and human (e.g. *Cricetulus griseus*, XP_003505818.1; *Homo sapiens*, NP_000910; *Rattus norvegicus*, NP_037132). Endogenous PAM activity is detectable in CHO cells, but levels are substantially below those found in professional secretory cells such as pituitary endocrine cells and atrial myocytes [8]. Consistent with the expression of PAM in CHO cells, many CHO cell monoclonal antibody preparations consist of mixtures of α-amidated and non-amidated product [5, 9–11]. We compared the ability of wildtype CHO cells and CHO cells expressing exogenous membrane or soluble PAM to secrete Fc-amidated peptide fusion proteins.

The growth hormone signal sequence was placed before the sequence of human Fc and a cleavable linker was placed between the C-terminus of Fc and the peptidylglycine substrate to facilitate product analysis. Three peptidylglycine substrates were selected for study. Peptide YY (PYY), which plays an important role in the response of the endocrine pancreas to oral glucose, terminates with -Tyr-NH_2_; amidation of its C-terminus is essential for bioactivity [12]. Neuromedin U (NMU), which is expressed in the gastrointestinal tract and brain and plays a major role in regulating energy balance, terminates with -Asn-NH_2_; amidation of its C-terminus is also essential for full bioactivity [13]. Glucagon-like peptide 1(GLP1), which stimulates pancreatic β cells to proliferate and release insulin in response to oral glucose, terminates with -Arg-NH_2_; its C-terminus is not essential for bioactivity [14, 15], but the high K_m_ values for –Arg-Gly peptides suggested that GLP1-Gly would be a good test case [16]. By placing the peptidylglycine substrate at the C-terminus of the Fc-fusion protein, any need for endo- or exoproteases in the biosynthetic pathway was eliminated.

## Results

### Design of vectors encoding Fc-amidated peptide precursors

**Fig. 1:**
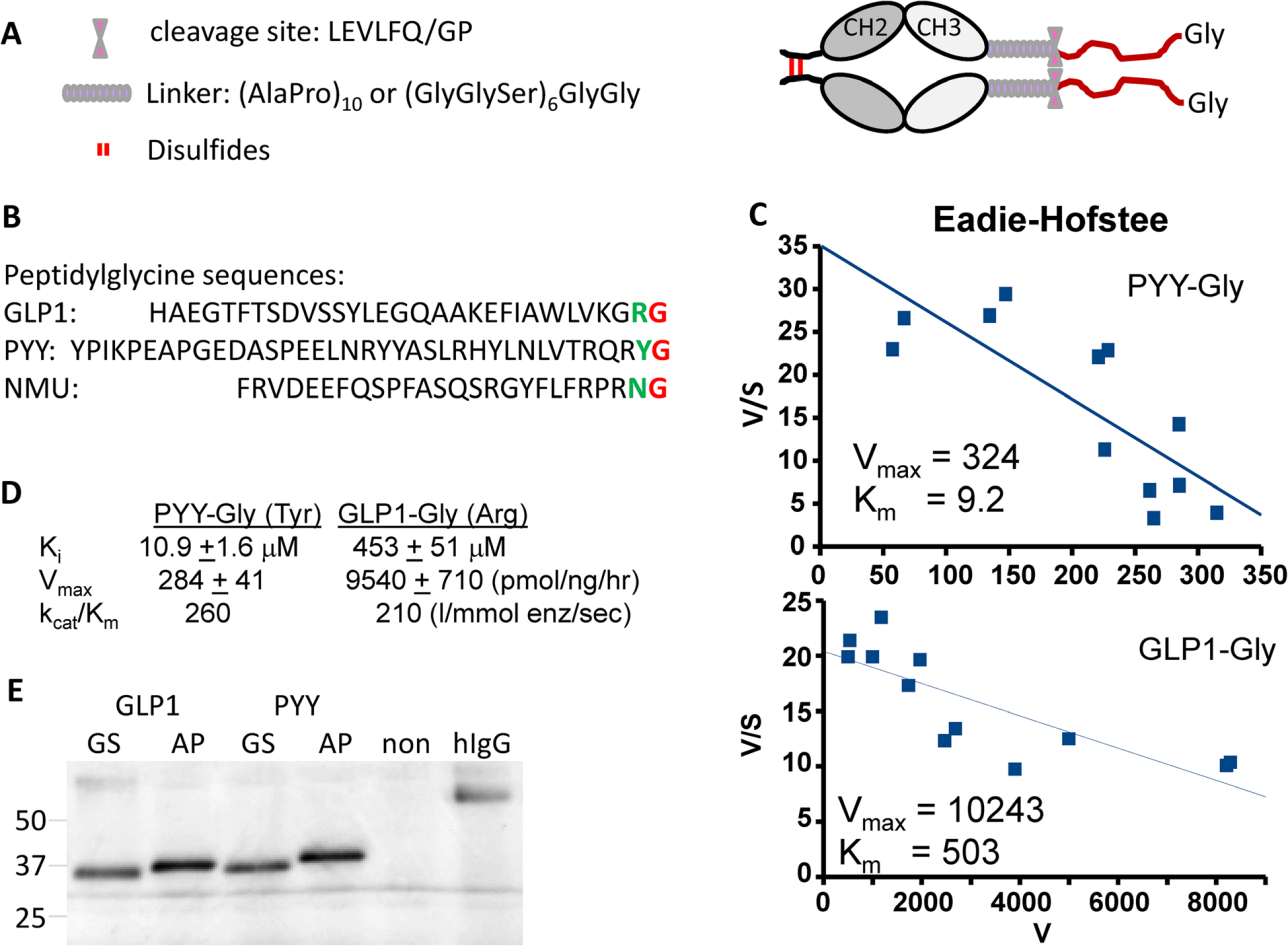
Design of vectors encoding Fc-peptidylglycine precursors. **a** Key features of the Fc-peptidylglycine precursors are illustrated. **b** The amino acid sequences of human GLP1-Gly, PYY-Gly and NMU-Gly are shown (http://www.bachem.com/research-products/). **c** Eadie-Hofstee plots illustrating the effects of synthetic PYY-Gly and GLP1-Gly on conversion of [^125^I]-Acetyl-Tyr-Val-Gly into [^125^I]-Acetyl-Try-Val-NH_2_ by purified PAM 820 s (0.085 ng/40 μl ≈ 1 fmol enzyme/tube). **d** Calculated kinetic parameters for PYY-Gly and GLP1-Gly. **e** pEAK Rapid cells were transiently transfected with vectors encoding Fc-GS-GLP1 (calculated mass discounting oligosaccharides; 31,720), Fc-AP-GLP1 (mass; 32,020), Fc-GS-PYY (mass; 32,730) and Fc-AP-PYY (mass; 33,090); control non-transfected cells (non) were analyzed at the same time. Aliquots of spent medium (20 μl) were fractionated by SDS-PAGE and visualized using antibody to human Fc; 18 ng human IgG (hIgG) was analyzed at the same time

The active site of PHM requires the presence of a C-terminal Gly; for most neuroendocrine peptide precursors, cleavage by an endoprotease followed by an exoprotease is required to generate the peptidylglycine substrate. To focus on the requirements for peptide amidation in the cellular environment, we appended the peptidylglycine substrates to the C-terminus of human Fc (Fig. 1a). The human growth hormone signal was used to ensure efficient protein entry into the secretory pathway. Use of two different linker peptides [(AP)_10_ or (GGS)_6_GG], was explored and a proteolytic cleavage site was inserted after the linker region so that the peptide product could easily be separated from the Fc-region, facilitating analysis of its amidation. Although PHM can accommodate any amino acid in the penultimate position, hydrophobic residues facilitate substrate binding [17]. To evaluate the effect of the penultimate amino acid on extent of amidation (the amidated residue is noted in parenthesis), we constructed vectors encoding GLP1-Gly (−Arg-NH_2_), NMU-Gly (−Asn-NH_2_) and PYY-Gly (−Tyr-NH_2_) (Fig. 1b).

The ability of GLP1-Gly and PYY-Gly to interact with PAM was assessed using synthetic peptides and purified soluble recombinant PAM (PAM820s). PAM activity was quantified using [^125^I]-Acetyl-Tyr-Val-Gly. The ability of synthetic GLP1-Gly and synthetic PYY-Gly to inhibit this reaction was assessed; data were plotted in Eadie-Hofstee format (Fig. 1c). While PYY-Gly had a K_i_ in the low μM range, the K_i_ of GLP1-Gly was about 50-fold higher. Predicted V_max_ values were also much higher for GLP1-Gly than for PYY-Gly, so that their catalytic efficiencies (k_cat_/K_i_) were similar (Fig. 1c and d).

Transient expression of the Fc-peptidylglycine fusion proteins was used to verify secretion of Fc proteins of the expected mass; secreted fusion proteins were detected using antibody to human Fc (Fig. 1e). Spent medium contained only the intact fusion proteins; there was no indication of degradation. The choice of linker had no effect on secretion of product, but the slightly greater mass of the (AP)_10_ linker [(AP)_10_, 1700 daltons; (GGS)_6_GG, 1139 daltons] was apparent.

### CHO lines used to express Fc-peptidylglycine fusion proteins

**Fig. 2:**
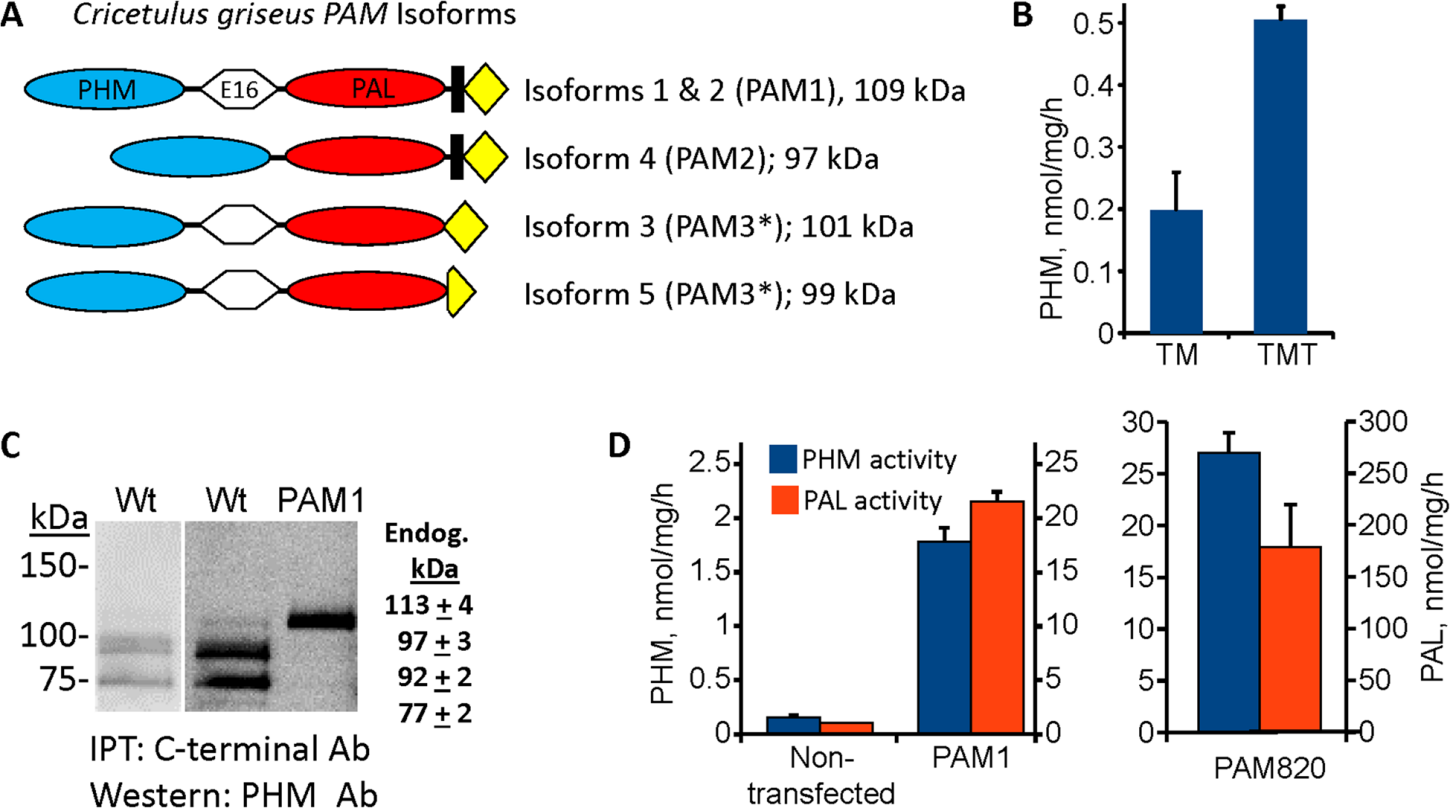
Characterization of endogenous CHO cell PAM. **a** Five isoforms of *Cricetulus griseus* PAM are included in the primary RefSeq assembly of the Chinese hamster genome; these isoforms closely resemble those observed in rat, mouse and human: XP_003505817.1, isoform 1; ERE82825.1, isoform 2; XP_003505819.1, isoform 3; XP_003505820.1, isoform 4; XP_003505821.1, isoform 5. **b** The specific activity of endogenous PHM in the soluble (TM) and solubilized (TMT) crude particulate fractions is indicated. **c** The solubilized particulate fraction from two separate preparations of wildtype CHO cells (224 μg protein) and from CHO cells stably expressing PAM1 (20 μg protein) were immunoprecipitated using an affinity-purified antibody to the C-terminus of PAM, eluted and fractionated by SDS-PAGE, transferred to PVDF membranes and visualized using affinity-purified antibody to the PHM domain (JH1761); molecular weights of marker proteins analyzed along with PAM1 are indicated. A lighter exposure is shown for the other Wt sample so that the doublet of 92 and 97 kDa bands is visible. Apparent molecular weights calculated from three separate analyses are shown ± Std Dev. **d** PHM and PAL assays were performed on lysates prepared from wildtype CHO cells and from CHO cells stably expressing rat PAM1 or rat PAM 820 s; lysates were prepared in 1 % TX-100

Earlier experiments documented the presence of PHM activity in CHO cell lysates [6] and the secretion of amidated peptides by CHO cells expressing exogenous peptide precursors [18]. However, the properties of CHO cell PAM have not been reported. Analysis of the *Cricetulus griseus* genome revealed the existence of transcripts corresponding to the major PAM splice variants detected in rat and human (Fig. 2a). While *Cricetulus griseus* isoforms 1, 2 and 4 would be integral membrane proteins, isoforms 3 and 5 would be soluble proteins. Isoforms 1 and 2 are products of alternative splicing at exon/intron junctions preceding and immediately following the transmembrane domain and differ in length by only three amino acids.

Endogenous PHM activity was detected in both crude particulate and soluble fractions prepared from CHO cells (Fig. 2b). PHM specific activity in the particulate fraction was 2.5 times higher than in the soluble fraction; 70 ± 5 % of the PHM activity in CHO cell lysates was particulate. The specific activity of PHM in CHO cell lysates was below levels observed in corticotrope tumor cells [19], making it impossible to characterize in crude extracts using existing antisera.

Immunoprecipitation was used to enrich PAM found in the TX-100 solubilized particulate fractions prepared from CHO cells (Fig. 2c). An affinity-purified antibody specific to the C-terminus of PAM, which is identical in mouse and Chinese hamster, was used to enrich CHO cell PAM. After separation by SDS-PAGE, antibody specific for the PHM region was used to determine the molecular weights of any cross-reactive proteins. For comparison, a PAM1 CHO cell lysate was immunoprecipitated at the same time. The minor 113 ± 4 kDa CHO cell protein recognized by the PHM antibody was also visualized by an antibody specific for exon 16 (not shown) and has the properties expected of isoforms 1 and 2 (PAM1). The closely spaced doublet of bands at 97 ± 3 kDa and 92 ± 2 kDa could represent Chinese hamster isoforms 3, 4 (PAM2) and 5; the lower band in the doublet is recognized by the exon 16 antibody. The 77 ± 2 kDa PAM protein is smaller than any of the characterized splice variants and may represent a cleavage product.

We next compared the level of PHM activity in wildtype CHO cells to the level present in CHO cells stably expressing PAM1 or PAM820s (Fig. 2d). Based on PHM specific activity, expression of PAM1 and PAM820s in the CHO lines used for expression of Fc-peptidylglycine fusion proteins was 10- and 100-fold higher, respectively, than endogenous PHM levels. In each line, PAL specific activity was approximately 10-fold higher than PHM specific activity, as expected [20].

### Characterization of Fc-peptidylglycine fusion proteins expressed by wildtype and PAM-expressing CHO cells

**Fig. 3:**
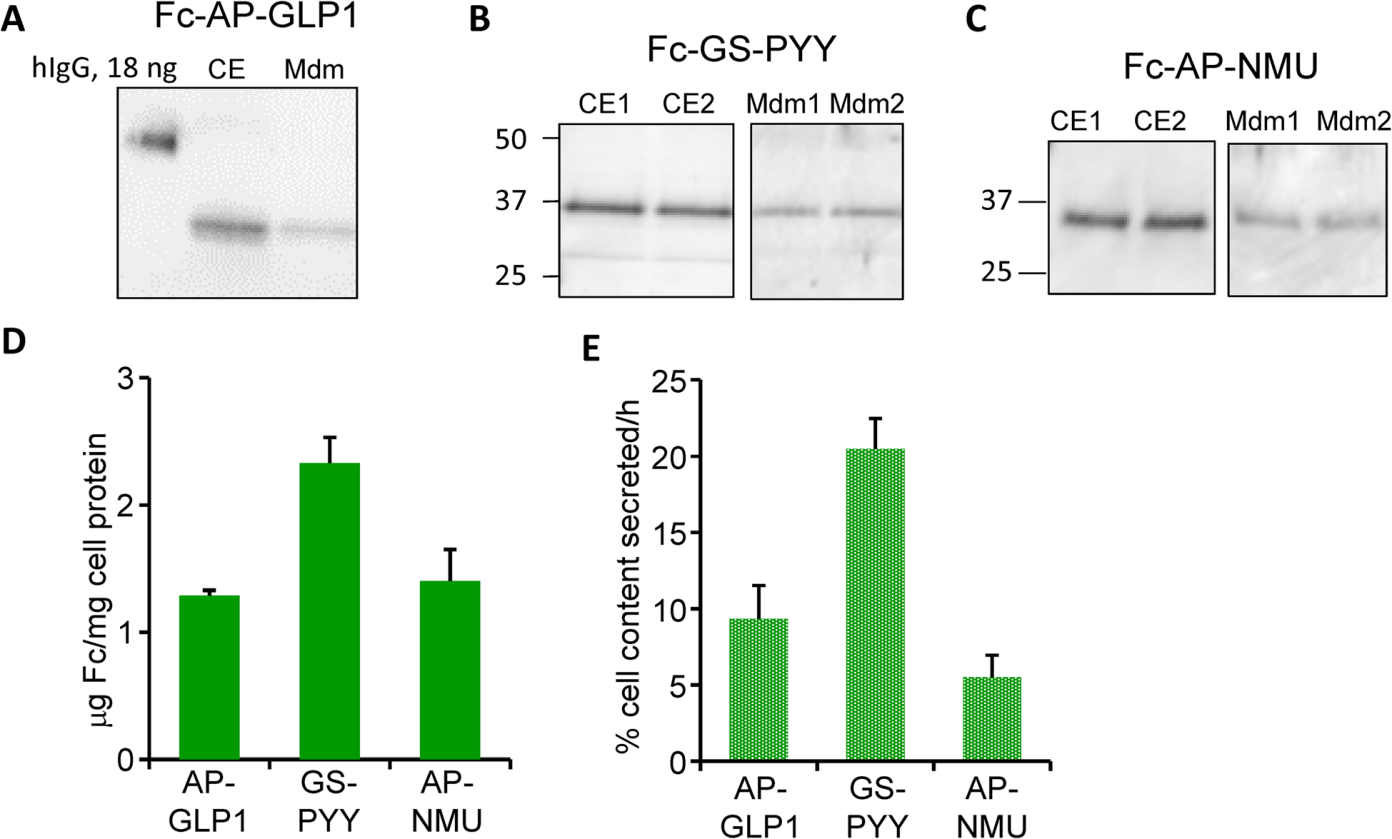
Characterization of Fc-fusion protein expression and secretion in wildtype CHO lines. Samples of cell extract (CE) and spent medium (16 h collection) prepared from CHO lines expressing Fc-AP-GLP1 (**a**) [15 μg (7 %) of CE; 0.9 % of spent medium], Fc-GS-PYY (**b**) [10 μg (8.5 %) of CE; 1.3 % of spent medium) and Fc-AP-NMU (**c**) [10 μg (5.3 %) of CE; 1.3 % of spent medium) were fractionated by SDS-PAGE; samples and a human IgG standard were visualized using antibody to human Fc. **d** Using GeneTools, data from several similar analyses (n = 3–4) were quantified to determine μg Fc/mg cell protein. **e** The secretion rate for Fc was calculated for several experiments by quantifying the amount of Fc recovered from the cell extract (CE) and from the medium using GeneTools; for each cell line, secretion rate was expressed as % cell content secreted per hour

We first examined the ability of stably transfected CHO cell lines to produce and secrete the three Fc-peptide fusion proteins; the amount of product recovered in spent medium was compared to the amount present in cell extracts (Fig. 3a-c). Fc content (μg Fc/mg protein) and secretion rate (% cell content/h) were quantified for each cell line. For Fc-GLP1 and Fc-NMU, fusion proteins containing both the AP and GS linkers were examined; no linker-related differences were apparent. For Fc-PYY, only the fusion protein with a GS linker was examined. Cell content of Fc was similar (1.3-2.6 μg Fc/mg cell protein) for all of the lines examined (Fig. 3d). The secretion rates varied from 5 % - 20 % of cell content per hour (Fig. 3e).

**Fig. 4:**
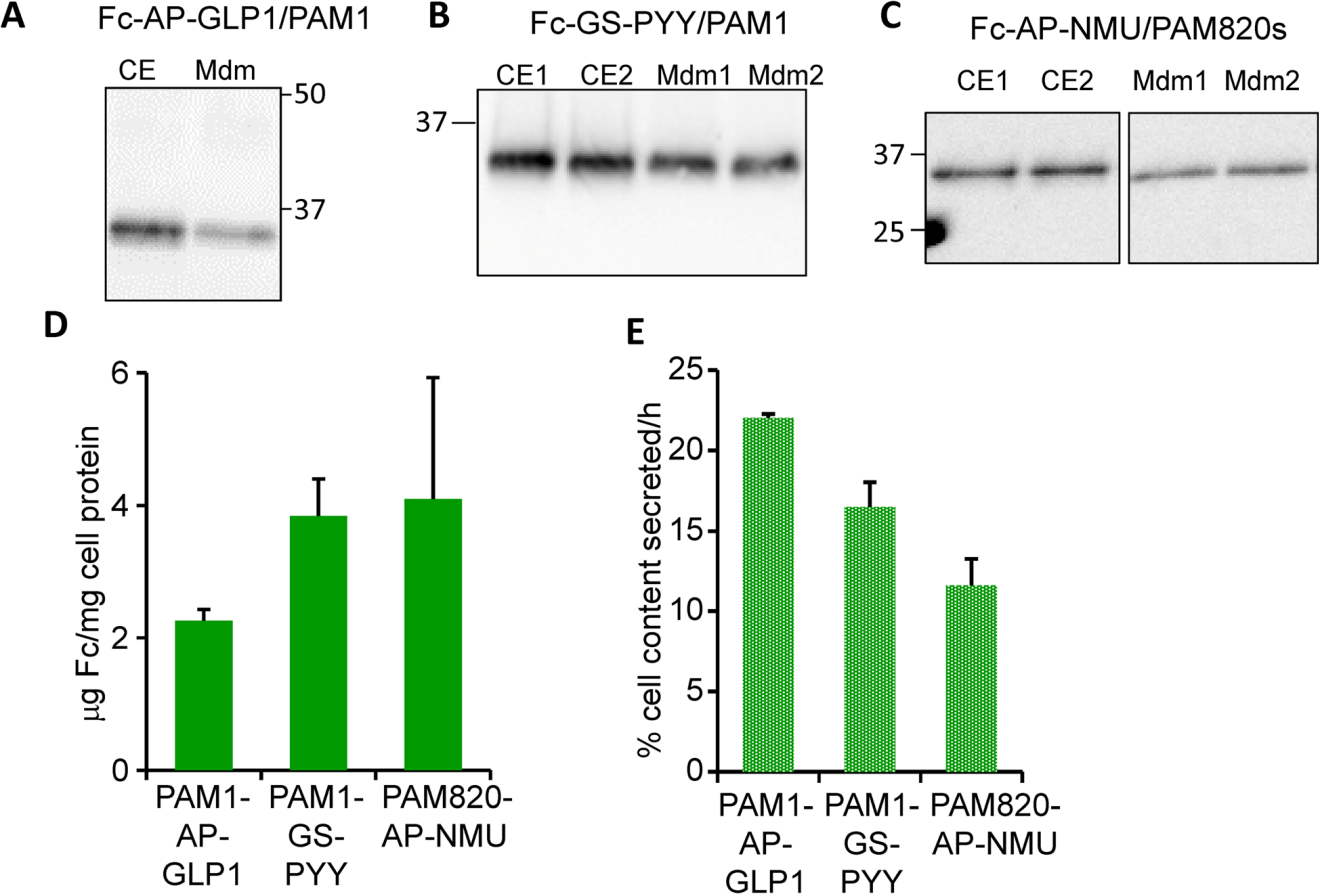
Characterization of Fc-fusion protein expression in CHO lines expressing PAM. Fc-AP-GLP1 (**a**) and Fc-GS-PYY (**b**) were expressed in PAM1 CHO cells; Fc-AP-NMU (**c**) was expressed in PAM820s CHO cells. Aliquots of cell extract (**a**, 5 %; **b**, 3 %; **c**, 6 % of total cell extract) and spent medium (**a**, 0.7 %; **b**, 1.3 %; **c**, 2.3 % of total medium volume) were prepared as described for Fig. 3. **d** Using GeneTools, data from several similar analyses were quantified to determine μg Fc/mg cell protein (**d**) and Fc secretion rate (**e**)

The same Fc-peptidylglycine fusion proteins were also expressed in CHO lines expressing exogenous PAM. Fc content and secretion rate were quantified for the PAM-expressing CHO cell lines in the same manner as for wildtype CHO cell lines (Fig. 4). Fc-GLP1 with the AP linker and Fc-PYY with GS linker were expressed in PAM1 CHO cells. Fc-NMU with both the AP and GS linkers was expressed in PAM820s CHO cells. Western blot analyses are shown (Fig. 4a-c), along with quantification of Fc content (Fig. 4d) and secretion rate (Fig. 4e). As for wildtype CHO lines, cell content of Fc ranged from 2–4 μg Fc/mg cell protein for all of the lines and Fc secretion rates were all in the range of 12-22 % of cell content per hour.

### Purification of secreted Fc-fusion proteins and evaluation of amidation status

**Fig. 5:**
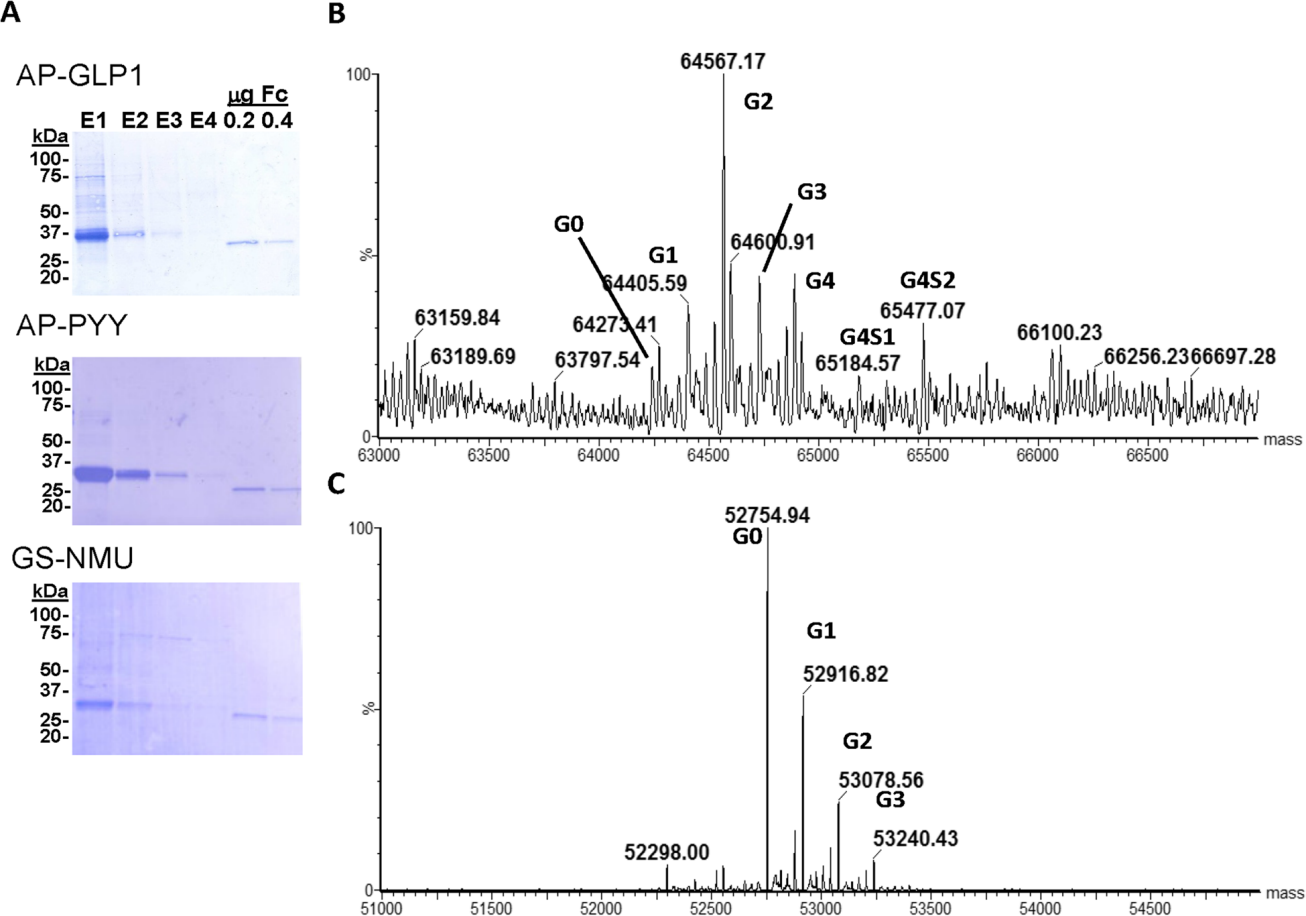
Purification and mass spectroscopic analysis of Fc-fusion proteins. **a** After concentration using a Vivaflow flip flow filtration system and a Centricon, secreted Fc-fusion proteins were purified by binding to HiTrap Protein A cartridges; bound protein was eluted by application of low pH buffer and aliquots (E1-E4) were subjected to SDS-PAGE; purified Fc was analyzed at the same time. Comparison of glycan heterogeneity by intact mass analysis of Fc-AP-GLP1 (**b**) and Fc from manufacturing cell line (**c**). Peak labels are shorthand notation for glycosylation state. All molecules exhibited two bi-antennary core fucosylated glycans (BiF/BiF) with an additional Gn (n = 1-4) galactose and Sn (=1–2) sialic acid (N-acetylneuraminic acid) moieties distributed between the two glycans

Monolayer cultures of stably transfected CHO lines were fed with complete serum-free medium every other day for a week; after concentration, spent media were applied to Protein A cartridges and bound Fc-fusion proteins were eluted with low pH buffer. SDS-PAGE analysis of the eluates, along with recombinant Fc, established their purity (Fig. 5a). Yields ranged from 1 to 5 mg purified Fc-fusion protein per liter of spent medium. Intact mass analysis of each product revealed the presence of a disulfide bonded dimer with a biantennary, core-fucosylated glycan. The observed glycosylation heterogeneity illustrated for the Fc-AP-GLP1 construct (Fig. 5b) was more diverse than is typically encountered in manufacturing CHO cell lines used for biotherapeutic (mAb) production, as exemplified by the Fc standard (Fig. 5c). The presence of some hybrid glycoforms would indicate that a low percentage of the Fc-fusion proteins were secreted prematurely (prior to full transit of the Golgi) or there was competition for substrate which interfered with glycosyltransferases.

**Fig. 6:**
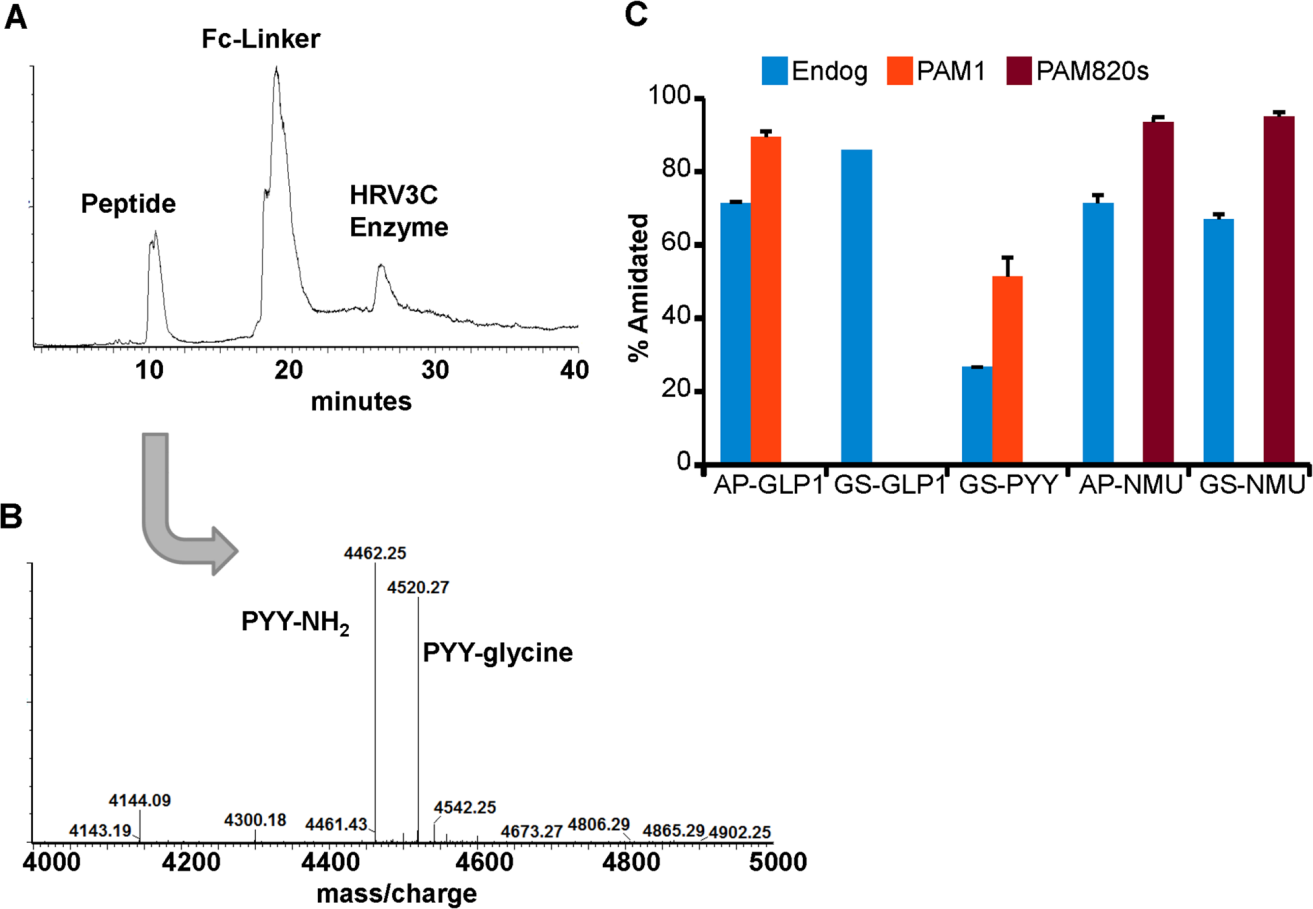
Reversed-phase HPLC-MS analysis of HRV3C cleavage products. **a** Total ion chromatogram (TIC); **b** single-charge deconvoluted mass spectrum (MaxEnt3) from the *m/z* data under the peptide peak. **c** Purified Fc-fusion proteins were cleaved with HRV3C protease and subjected to mass spectroscopic analysis as described in Methods. Each sample was analyzed in triplicate; standard errors are shown

Intact mass analysis was not precise enough to determine the degree of C-terminal amidation. Therefore, extent of amidation was quantified by analysis of the HRV3C rhinovirus protease cleavage products (Fig. 6a). Peptidylglycine precursors differ from their amidated products by a mass of +58; amidated peptides differ from the corresponding peptide with a free COOH-terminus by a mass of −1 and a charge change (+1) at neutral pH. Intact mass analysis of the cleaved proteins allowed reliable identification of amidated product and glycine-extended precursor (Fig. 6b). Product lacking the COOH-terminal Gly, with a free COOH-terminus was not identified. Some off-target cleavage by rhinovirus protease was observed; instead of the expected cleavage between Q and G^248^ (LEVLFQ/GP), cleavage occasionally occurred before F^246^ or Q^247^. Each purified product was analyzed in triplicate. For each substrate, the percent amidated was calculated by summing amidated plus glycine-extended peptide to determine the total amount of peptide recovered, and assumes there is no difference in HRV3C specificity or cleavage rate between the two substrates.

Fc-GLP1 was almost 90 % amidated, even when expressed in wildtype CHO cells having only endogenous levels of PAM (Fig. 6c). No difference in extent of Fc-GLP1 amidation was observed in CHO cells expressing PAM1. In contrast, less than 30 % of the Fc-PYY produced by wildtype CHO cells was amidated; expression of PAM1 increased the extent of PYY amidation by about a factor of two (Fig. 6c). Approximately 71 % of the Fc-NMU produced by wildtype CHO cells was amidated; Fc-AP-NMU and Fc-GS-NMU did not differ in their extent of amidation. Fc-NMU produced by CHO cells expressing soluble PAM (PAM820s) was over 90 % amidated, a significant increase over the extent of amidation observed in wildtype CHO cells.

### Assessment of biological activity

The peptidylglycine substrates tested each yield amidated peptides that interact with specific GPCRs. We therefore used cell-based receptor assays to test the biological activity of two of the purified secreted Fc-fusion proteins. The concentration of each Fc-fusion protein was determined by comparison to a standard curve generated using purified human Fc analyzed on the same Western blot.

**Fig. 7:**
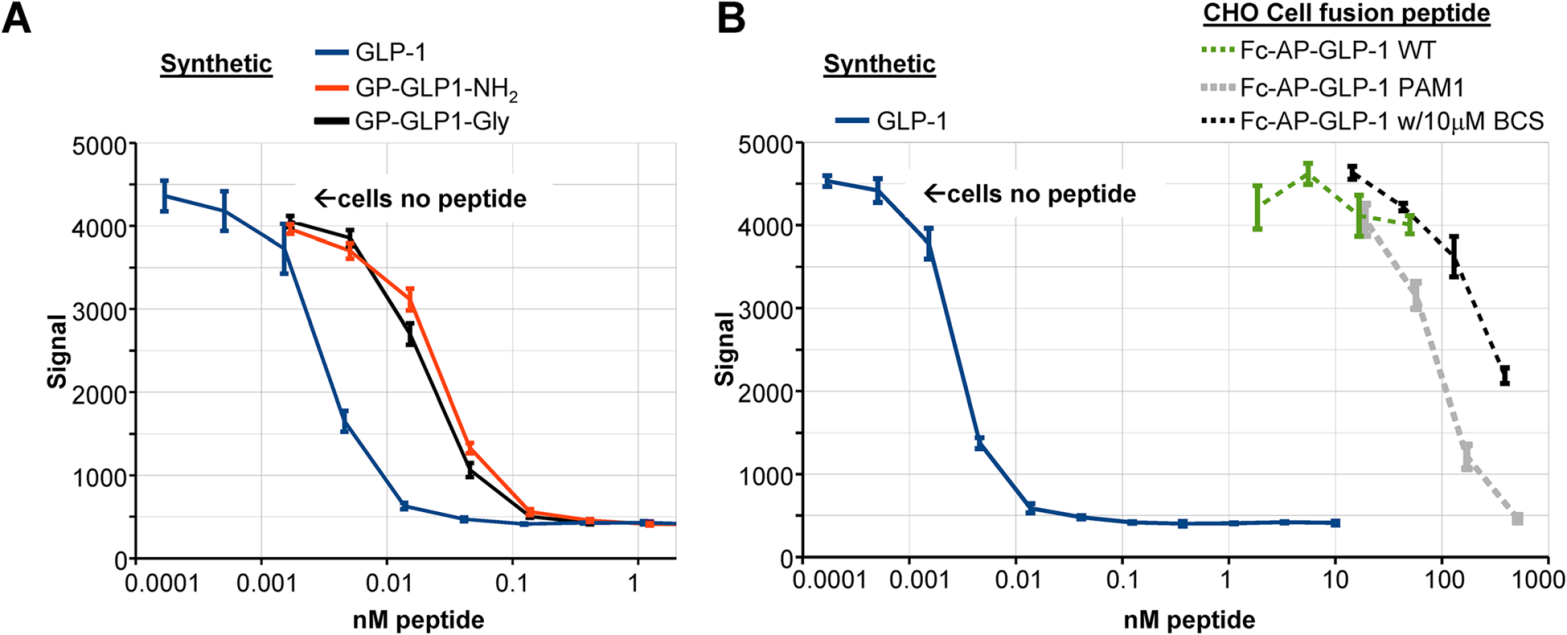
Bioassay of purified Fc-GLP1 fusion proteins. HEK cells expressing the GLP-1R were used to assess the ability of Fc-GLP1 fusion proteins purified from the spent medium of different CHO cell lines to stimulate cAMP production. **a** The activity of GLP1, GP-GLP1-NH_2_ and GP-GLP1was compared; as expected, amidation did not affect bioactivity while appending the GP dipeptide to its amino-terminus reduced the potency of GLP1-NH_2_. **b** The activity of GLP1 was compared to that of Fc-AP-GLP1 fusion proteins purified from the spent media of WT CHO cells, CHO cells expressing PAM1 and WT CHO cells treated with 10 μM bathocuproine disulfonate to inhibit endogenous PHM throughout the entire collection period. Parallel dose–response curves were observed for all of the peptides and proteins tested; Fc-GLP1 was at least 10,000-fold less active than GLP1. The y-axis shows the TR-FRET signal at 665 nm; the signal obtained by cells in the absence of any added peptide is indicated

The bioassay for GLP1 used HEK cells expressing the GLP-1R; synthetic GLP1-Ser8 was used as the standard and had an EC_50_ of 2.8 pM (Fig. 7a). The NH_2_-terminus of GLP1 plays an essential role in its interaction with receptor and the addition of the GP dipeptide to the NH_2_-terminus of synthetic GLP1 increased its EC50 to 25.8 pM (Fig. 7a). The corresponding glycine-extended peptide had a similar EC50, as expected for a ligand/receptor interaction that is known not to require the COOH-terminus of the ligand [14, 15]. Although the intact Fc-GLP1 fusion proteins tested yielded dose response curves that were parallel to those of GLP1, they were at least 10,000-fold less potent than GLP1; their EC_50_ values were in the 100 nM range (Fig. 7b).

**Fig. 8:**
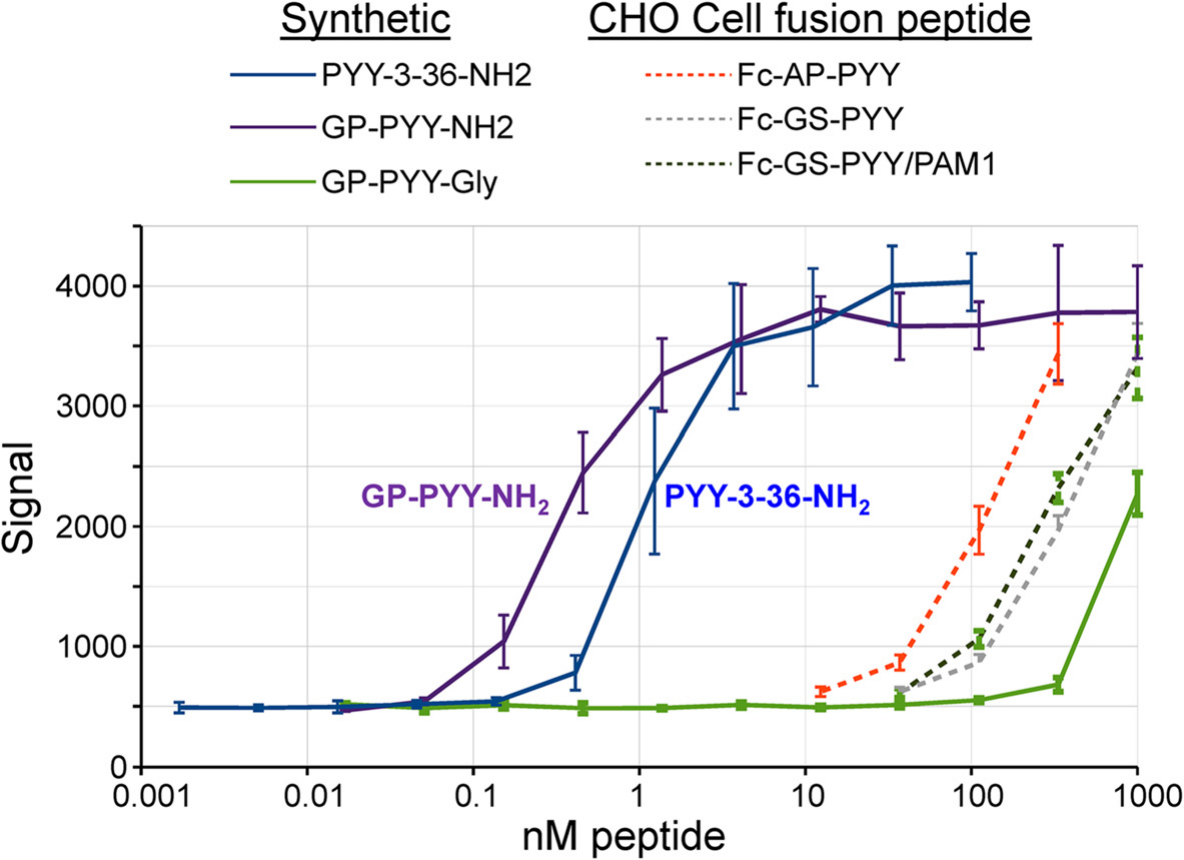
Bioassay of purified Fc-PYY fusion proteins. CHO cells expressing the NPY-2R were used to assess the ability of Fc-PYY fusion proteins purified from the spent medium of different CHO lines to inhibit forskolin-stimulated cAMP production; PYY binding to NPY-2R activates G_αi_, decreasing cAMP production. Synthetic PYY(3–36)NH_2_ was more potent than synthetic GP-PYY-NH_2_; synthetic GP-PYY-Gly was at least 10,000-fold less potent than the corresponding amidated peptide. Although the Fc-PYY fusion protein produced by PAM1 cells was about 200-fold less active than synthetic GP-PYY-NH_2_, it was 10-fold more active than synthetic GP-PYY-Gly. Parallel dose–response curves were observed for all of the peptides and proteins tested

The bioassay for PYY used CHO cells expressing the NPY-2R; synthetic PYY(3–36)NH_2_ was used as the standard and had an IC_50_ of 1.2 nM (Fig. 8). Synthetic peptides corresponding to full-length PYY with the GP dipeptide left by rhinovirus protease cleavage of the Fc-PYY substrate were used to evaluate the effect of COOH-terminal amidation. GP-PYY-NH_2_ was slightly more potent than PYY(3–36)NH_2_ while GP-PYY-Gly was 1000-fold less potent (Fig. 8). The Fc**-**PYY products examined yielded dose response curves that were parallel to those of PYY(3–36)NH_2_ and GP-PYY-NH_2_. Although their IC_50_ values were at least 100-fold higher than the synthetic peptide, the Fc-PYY products were 10-fold more potent than GP-PYY-Gly.

## Discussion

CHO cells have served as excellent mammalian cell factories for making proteins that are difficult or impossible to create in bacterial and yeast systems [5]; the α-amidating enzyme, PAM, falls into this category and has been successfully expressed in CHO cells [21–24]. Thus, to produce large, α-amidated proteins for therapeutic or investigational purposes, CHO cells were selected. The plasma half-life of many peptides has been increased several orders of magnitude by fusion with the Fc region of human immunoglobulin [1], making the Fc-propeptide fusion a desirable starting point to make long-lived proteins terminating with the α-amidated peptide of interest.

Although the specific activity of endogenous CHO cell PAM is low compared to endocrine cells or atrial myocytes [25], it is clearly capable of α-amidating exogenous proteins. CHO cells expressing monoclonal antibodies that terminate with a C-terminal Gly residue are often partially α-amidated [5, 9]. The ability of endogenous CHO cell PAM to α-amidate a significant amount of each Fc-peptidylglycine precursor is consistent with the properties of PAM established using test tube assays and the levels of enzyme and substrate expressed. When assayed with substrate levels well below the K_m_ of the enzyme (0.5 vs. 9.2 μM; Fig. 1), non-transfected CHO cells had a specific activity of 0.1 nmol/mg protein/h (Fig. 2); based on Michaelis-Menten kinetics and allowing for the molecular weight of the fusion protein, this corresponds to a maximal amidation rate of 45 μg Fc-peptide/mg protein/h. Since the cell lines studied here contained only 1–3 μg Fc-peptide/mg protein and secreted only 5 to 20 % of their cell content per h (Fig. 3), they would be expected to α-amidate a significant fraction of the secreted Fc-peptide. Cell lines producing significantly more Fc-peptide may need exogenous PAM and increased storage capacity to produce more extensively amidated product.

Comparison of cell-based and test tube assays suggests that PAM is operating at close to V_max_ in cells – its affinity for the Fc-peptidylglycine substrate is not a major factor. GLP1-Gly, with a penultimate Arg residue, was more extensively amidated than PYY, with a penultimate Tyr residue (Fig. 6c). By comparison, in test tube assays, peptides ending in –Aromatic-Gly are better substrates than peptides ending in –Charged-Gly [16]. The more rapid secretion of Fc-PYY than Fc-GLP1 (Fig. 3e) may contribute to this phenomenon, although an explanation for this difference is not apparent. The very high concentration of protein in the lumen of the secretory pathway (over 100 mg protein/ml) is difficult to mimic in test tube assays [26, 27].

Both the cellular machinery and substrate will need to be optimized in order to achieve the maximal yield of extensively α-amidated product. Clearly the rate of secretion of the Fc-fusion peptide (Fig. 3) has a role, since the Fc-peptides secreted slowly (GLP1 and NMU: 5-9 % of cell content/h; Fig. 3) were highly amidated without the need for additional PAM (Fig. 6c). By contrast, the cells which secreted the fusion peptide rapidly (PYY; Fig. 3) showed less extensive α-amidation of the peptide (25 %; Fig. 6c) and the extent of amidation was improved by expression of exogenous PAM (50 %; Fig. 6c). Fc-PYY provides a better test case for assessing modifications aimed at increasing the extent of α-amidation than Fc-GLP1.

## Conclusions

The ultimate goal of this work is creation of long-lived peptides for therapeutic or investigational uses. The peptides chosen for this initial study were selected to answer specific questions about the methodology and the ability of PAM to handle different substrates. The limited ability of Fc-PYY-NH_2_ to activate the NPY-2R may reflect the role of the N-terminal region of PYY in its interaction with receptor [28] or interference by the Fc-region with access of PYY-NH_2_ to its receptor. A different carrier protein might better confer stability to the fusion protein without compromising receptor accessibility. The properties of the carrier protein may also determine the speed with which it traverse the secretory pathway and the time over which it is exposed to PAM. The properties of PAM are unlikely to limit the choice of carrier protein. Fusion protein stability will need to be assessed; the ability of purified Fc-PYY to activate the NPY-2R may reflect cleavage during the incubation with cells. It is not currently feasible to attempt to recover the peptide after the bioassay, and any cleaved peptide that was endocytosed by the reporter cells would also be missed. Whether using their endogenous enzyme or exogenous PAM, CHO cells can efficiently amidate large proteins that terminate with a Gly residue.

## Methods

### Expression vector design

Two linker peptides were used: AP, (Ala-Pro)_10_; GS, (Gly-Gly-Ser)_6_-Gly-Gly. Expression vectors encoding Fc-AP-GLP1-Gly, Fc-GS-GLP1-Gly, Fc-AP-NMU-Gly, Fc-GS-NMU-Gly, Fc-AP-PYY-Gly and Fc-GS-PYY-Gly were synthesized at Genewiz, Inc. (South Plainfield, NJ) and cloned into an expression vector with a blasticidin selectable marker. The GH signal sequence preceded human IgG1(CPPCPA---LSLSPG). A 20 residue AP linker [(AP)_10_-LEVLFQ/GP] or GS linker [(GGS)_6_GG- LEVLFQ/GP] was appended to residue 475 of human IgG1 (AAA02914.1); cleavage at the HRV3C human rhinovirus protease site adds a Gly-Pro to the N-terminus of the product peptide.

### Generation of cell lines

In addition to wildtype CHO DG44 cells, we selected CHO lines expressing PAM1 and PAM820s encoded by a pCis vector, using selection in αMEM lacking nucleotides and nucleosides [29]. For cells transfected with vectors conferring blasticidin resistance, growth medium was a 1:1 mixture of Dulbecco’s Modified Eagle’s Medium:Ham’s F-12 [Lonza, Allendale, NJ] containing 25 mM Hepes, 50 IU/ml Penicillin, 50 μg/ml streptomycin [Cellgro, Manassas, VA], 10 % fetal bovine serum [Hyclone, Waltham, MA] and 3 μg/ml blasticidin [InvivoGen, San Diego, California]. For cells transfected with pCis vectors, growth medium was αMEM [Cellgro, Manassas, VA] containing 25 mM Hepes, 50 IU/ml Penicillin, 50 μg/ml streptomycin, 10 % dialyzed fetal bovine serum and 2 mM L-glutamine [Cellgro]. Doubly transfected cells were maintained in the pCis selection medium with 3 μg/ml blasticidin. Cells were passaged using trypsin-EDTA [Cellgro].

CHO DG44 cells grown to 50 % confluence in T75 flasks [BD Falcon, Franklin Lakes, New Jersey] were rinsed with 20 ml of serum free medium for 30 min prior to transfection. Cells were transfected using 30 μg plasmid DNA, 60 μl Lipofectamine incubated in 200 μl Opti-MEM [Invitrogen, San Diego, California] and diluted into 5 ml Opti-MEM before application onto the cells; transfection solution was removed after 7 h and cells were fed with the appropriate growth medium containing 3 μg/ml blasticidin. To screen for Fc-peptide secretion, single colonies in a 96-well plate [Corning, Manassas, VA] were fed with 80 μl CSFM; spent medium was harvested the next morning, and 20 μl was fractionated by SDS-PAGE on a 10 lane, 4-15 % Tris–HCl Precast gel [Bio Rad, Hercules, CA]. Secreted product was visualized using rabbit anti-human Fc [309-001-008; Jackson Immunoresearch, West Grove, PA]. Selected single colonies were trypsinized and cells were transferred into one well of a 12-well plate [Corning, Manassas, VA]. After the cells had grown for several days, they were trypsinized so that one aliquot could be frozen and another aliquot subcloned again using a 96-well plate. Subcloning was repeated until all of the single colonies tested were positive for Fc secretion and a colony was selected for use.

### Analysis of Fc-peptide fusion protein and PAM expression and secretion

Cells were grown to confluence in 6- or 12-well dishes. Secretion rates for Fc-peptide fusion proteins and for PAM were determined by incubating cells in complete serum free medium (CSFM) containing 25 mM Hepes, 50 IU/ml Penicillin, and Insulin-Transferrin-Selenium [Invitrogen, San Diego, California] [3, 4]. Spent medium was collected, centrifuged to remove any cell debris and stored frozen after addition of protease inhibitors [30]. For assessment of PHM and PAL activity in cell lysates, cells were extracted into ice cold 20 mM TES, 10 mM mannitol, titrated to pH 7.4 with NaOH (TM), or with added 1 % TX-100 (TMT) (SurfActs, Pierce, Waltham, MA) containing PMSF and protease inhibitors [30]; extracts were frozen and thawed 3 times, centrifuged at 14,000 x g for 15 min, and supernatants were stored in aliquots at -80C. For evaluation of Fc-peptide fusion protein secretion, cells were extracted by heating for 5 min at 95 °C in SDS lysis buffer.

Cell lysate protein concentrations were determined using the BCA Assay with bovine serum albumin as the standard [Pierce, Waltham, MA]. Samples for SDS-PAGE were diluted into 1X or 5X Laemmli sample buffer, heated for 5 min at 95 °C and loaded onto 4-15 % Tris–HCl 12 + 2 well Criterion Precast Gels [Thermo Scientific, Waltham, MA]. A high molecular weight Precision Plus standard [Bio-Rad, Hercules, CA] was analyzed each time. Electrophoresis was carried out for 90 min at 150 V; proteins were transferred onto Immobilon PVDF membranes for 120 min at 300 mAmp. Membranes were blocked by incubation in 5 % fat-free powdered milk in 50 mM Tris·HCl, 150 mM NaCl, pH 7.5 containing 0.1 % Tween-20 (TTBS) for 1 h at RT [6, 7]. Primary antibodies were diluted into TTBS. Incubation with primary antibody was carried out at 4° C overnight. Membranes were washed 3X in TTBS and HRP-tagged secondary antibody diluted into TTBS was then added. Blots were incubated in secondary antibody for 2 h at RT and then washed 3X in TTBS and 1X in TBS. SuperSignal West Pico Chemiluminescent Substrate [Pierce, Rockford, IL] was added for 5 min at RT, and signals were visualized using a GeneGnome digital imaging system [Syngene, Frederick, MD]. GeneTools software [Syngene, Frederick, MD] was used for quantification; exposure times were selected to avoid saturation. A rectangle of equal size was used to identify each band; the automatic background correction was utilized. The signal for each sample was converted into pmol IgG or Fc using a conversion factor calculated from the human IgG or Fc standard analyzed on the same blot.

Purified human IgG [I4506, Sigma, St. Louis, MO] was dissolved in PBS; based on the A_280_ of a 10-fold dilution of this stock, its concentration was 7.3 mg/ml (extinction coefficient at 280 nm for human IgG 1.40; Thermo Scientific TechTip #6). Further dilutions were made so that 54 ng and 18 ng of hIgG was loaded onto a gel; 1 ng = 20 fmol monomer. Purified recombinant human Fc from papain digestion of commercially available immunoglobulin was also used as a standard for SDS-PAGE; based on the A_280_ of a 10-fold dilution of this stock, its concentration was 30.6 mg/ml (extinction coefficient at 280 nm for human Fc, 1.334). Further dilutions were made so that 25 ng, 10 ng, and 5 ng of human Fc was loaded onto a gel; 1 ng = 28.6 fmol monomer.

### Enzyme assays

Samples of spent media or cell extracts were diluted into PHM diluent (20 mM NaTES, pH7.4, 10 mM mannitol, 1 % TX-100 [SurfActs, Pierce, Rockford, IL], 1.0 mg/ml bovine serum albumin). The 40 μl reaction mixture contained a trace amount of [^125^I]-Ac-Tyr-Val-Gly, 2 μM CuSO_4_, 0.5 mM ascorbate, catalase, and 0.5 μM AcTyrValGly diluted into 150 mM NaMES, pH 5.5 [6]. Samples were assayed in duplicate at two dilutions and data were averaged. For PAL assays, each tube contained a trace amount of [^125^I]-Ac-Tyr-Val-α-hydroxylglycine, 0.5 μM Ac-Tyr-Val-α-hydroxylglycine, 0.05 % NP-40 and 1 mM CdCl_2_ diluted into 150 mM NaMES, pH 5.5 [7]. Samples were assayed in duplicate and data were averaged; assays typically contained 0.02 μg to 1.4 μg cell protein.

The ability of synthetic peptidylglycine peptides to inhibit the amidation of Ac-Tyr-Val-Gly was assessed by adding increasing concentrations of competitor to the assay mix. The PYY-Gly peptide (MW 4522) and the GLP1-Gly peptide (MW 3509) each included an N-terminal Gly-Pro dipeptide, to mimic the product expected from rhinovirus protease cleavage. Peptides were synthesized by New England Peptide (Gardner, MA) and were >99 % pure based on RP-HPLC/MS analyses performed in-house.

### Analysis of endogenous CHO cell PAM

Wildtype CHO cells were rinsed with CSFM and cell pellets were extracted by freeze/thaw cycles in 20 mM NaTES, 10 mM mannitol, pH 7.4 (TM); lysates were then centrifuged to separate the soluble fraction from the crude particulate fraction. Proteins in the crude particulate fraction were solubilized in TMT. Endogenous PAM recovered in the solubilized particulate fraction was immunoprecipitated using affinity-purified antibody to the C-terminus of PAM (0.5 μg C-STOP antibody for 90 to 230 μg CHO cell protein) [31]. PAM/antibody complexes were bound to Protein A Sepharose beads and eluted by incubation in Laemmli sample buffer at 95 °C. Eluates were fractionated by SDS-PAGE and CHO cell PAM proteins were visualized using affinity-purified antisera to PHM, Exon 16 and CD.

### Purification of recombinant Fc-peptide products

Individual Hyperflasks [Corning, Manassas, VA] were seeded with cells (11 to 45 × 10^6^ cells) from eight confluent T75 flasks suspended in 565 ml of growth medium. When the cells were 75-100 % confluent, they were washed for 30 min with 515 ml CSFM; the wash medium was discarded and fresh CSFM was added. Spent medium was harvested after 48 h and replaced with fresh CSFM; a total of four collections were made over a 7 day period. Proteins in the spent medium were concentrated in two steps. The volume was reduced from the 2 l obtained per Hyperflask to about 115 ml using a Vivaflow 200 with a cartridge that had a 30 kDa MWC polyethersulfone membrane [05VF20026; VivaScience, Littleton, MA]; the sample was equilibrated with 20 mM NaHPO_4_, pH 7.0 (Binding Buffer) [29]. Ultra-15 Centrifugal Filters [30,000 MWCO; Millipore, Billerica, MA] equilibrated with binding buffer were used to reduce the volume to ~2 ml.

Final purification was accomplished using Protein A HP SpinTrap columns [600 μl; GE Healthcare, Fairfield, CT] equilibrated with 600 μl Binding Buffer (20 mM sodium phosphate, pH 7.0). Aliquots of the Centricon concentrate were allowed to bind sequentially until the entire 2 ml sample had been added. The column was then washed five times with 600 μl Binding Buffer. Elution Buffer (400 μl, 0.1 M glycine/HCl, pH 2.7) was allowed to incubate with the cartridge for 1 min before centrifugation into 30 μl neutralizing buffer (1 M Tris–HCl, pH 9); this process was repeated four times. Input and eluates were analyzed by SDS-PAGE. Purified product concentration was determined by measuring A_280_ and using the extinction coefficient for human Fc.

### Mass spectroscopic analysis of purified Fc-peptide products

Initial analysis of Fc-peptide products was carried out by reversed-phase HPLC/MS of the intact or reduced (5 mM DTT, 55 °C, 30 min) product on the system described below. However, mass accuracy and measurement precision were insufficient to ensure unambiguous determination of the state of the peptide carboxy terminus. Consequently, samples were digested with HRV3C protease (PreScission, GE Healthcare Bio-Sciences, Pittsburgh, PA). Digestions of Fc-peptide were performed for 5 h at 5 °C in a buffer consisting of 20 mM TrisHCl, pH 7.0, 150 mM NaCl, 1 mM DTT using one unit of enzyme for up to 100 μg of protein. Polypeptides resulting from the digestion were chromatographically resolved on a Waters (Milford, MA) Acquity UPLC with an Agilent (Santa Clara, CA) PLRP-S column (2.1 × 50 mm, 5 μ d_p_, 1000 Å) using a multi-linear gradient. Buffer A was 0.1 % formic acid, and buffer B was 0.1 % formic acid in acetonitrile. The peptides were mass analyzed on a Waters API US mass spectrometer scanned from *m/z* 500–3000 at a rate of 1 Hz. The peptide *m/*z data was deconvoluted to the single-charge mass domain by the MaxEnt3 algorithm implemented in the Waters MassLynx data system.

### Functional bioassay for analysis of recombinant Fc-peptide products

A cell based homogeneous time-resolved fluorescence resonance energy transfer competitive immunoassay was used to quantify bioactive recombinant Fc-fusion proteins. Activation of a G_s_ or G_i_ coupled receptor results in an increase or decrease, respectively, in intracellular cAMP levels, which were quantified using the LANCE ultra cAMP assay kit (Perkin Elmer, Waltham, MA). The G_s_-coupled human GLP-1R stably expressed in HEK cells (in house) was used to quantify GLP-1 related peptides. The G_i_-coupled NPY-2R stably expressed in CHO cells (DiscoveRx, Freemont, CA) was used to quantify PYY related peptides. In order to detect agonist-induced reductions in cAMP levels in NPY-2R expressing cells, they were stimulated with forskolin (3 μM) to increase the intracellular cAMP.

Assays were performed in 384-well Opti-plates. On the day of the experiment, vials of frozen cells were thawed in a 37 °C water bath; cells were transferred into a tube containing PBS, filtered through a BD Falcon Cell strainer and counted. Cells were pelleted and suspended at a concentration of 0.5 × 10^6^ cells/ml in HBSS containing 5 mM HEPES and 0.1 % BSA. After cells were allowed to recover at room temperature for at least one hour, Fc-GLP1 and Fc-PYY fusion proteins were prepared in the same buffer without or with 6 μM foskolin, respectively; three-fold serial dilutions were made and cells (10 μl; with 0.5 mM IBMX added after recovery) and peptide (10 μl) were added to each well. Synthetic peptides were assayed along with the purified Fc-proteins. Covered plates were incubated at room temperature with gentle rotation on a plate shaker (~400 rpm) for 30 min. LANCE cAMP detection reagent mix (20 μl/well) (Cat# TRF 0264) was added to each well and allowed to incubate, with shaking, at room temperature for 1 h. Finally, signal was read on a Perkin Elmer Envision plate reader using the manufacturer’s recommended protocol.

### Animal ethics

This work used no animals, only established cell lines.

## Abbreviations

CHO: Chinese hamster ovary
GLP1: Glucagon-like peptide 1
NMU: Neuromedin U
PAL: Peptidyl-α-hydroxyglycine α-amidating lyase
PAM: Peptidylglycine α-amidating monooxygenase
PYY: Peptide YY

## References

[1] Czajkowsky DM, Hu J, Shao Z, Pleass RJ (2012). Fc-fusion proteins: new developments and future perspectives. EMBO Mol Med.

[2] Kulathila R, Merkler KA, Merkler DJ (1999). Enzymatic formation of C-terminal amides. Nat Prod Rep.

[3] Prigge ST, Mains RE, Eipper BA, Amzel LM (2000). New insights into copper monooxygenases and peptide amidation: structure, mechanism and function. Cell Mol Life Sci.

[4] Czyzyk TA, Ning Y, Hsu M-S, Peng B, Mains RE, Eipper BA, Pintar JE (2005). Deletion of peptide amidation enzymatic activity leads to edema and embryonic lethality in the mouse. Dev Biol.

[5] Skulj M, Pezdirec D, Gaser D, Kreft M, Zorec R (2014). Reduction in C-terminal amidated species of recombinant monoclonal antibodies by genetic modification of CHO cells. BMC Biotechnol.

[6] Kolhekar AS, Keutmann HT, Mains RE, Quon ASW, Eipper BA (1997). Peptidylglycine α-hydroxylating monooxygenase: active site residues, disulfide linkages, and a Two-domain model of the catalytic core. Biochemistry.

[7] Kolhekar AS, Bell J, Shiozaki EN, Jin L, Keutmann HT, Hand TA, Mains RE, Eipper BA (2002). Essential features of the catalytic core of peptidyl-alpha-hydroxyglycine alpha-amidating lyase. Biochemistry.

[8] Braas KM, Stoffers DA, Eipper BA, May V (1989). Tissue specific expression of rat peptidylglycine α-amidating monooxygenase activity and mRNA. Mol Endocrinol.

[9] Tsubaki M, Terashima I, Kamata K, Koga A (2013). C-terminal modification of monoclonal antibody drugs: amidated species as a general product-related substance. Int J Biological Macromolecules.

[10] Kaschak T, Boyd D, Lu F, Derfus G, Amanullah A, Yan B (2011). Characterization of the basic charge variants of a human IgG1: effect of copper concentration in cell culture media. MAbs.

[11] Johnson KA, Paisley-Flango K, Tangarone BS, Porter TJ, Rouse JC (2007). Cation exchange-HPLC and mass spectrometry reveal C-terminal amidation of an IgG1 heavy chain. Anal Biochem.

[12] Keire DA, Bowers CW, Solomon TE, Reeve Jr JR. Structure and receptor binding of PYY analogs. Peptides. 2002;305–321. 10.1016/s0196-9781(01)00602-7 11825645

[13] Brighton PJ, Szekeres PG, Willars GB (2004). Neuromedin U and its receptors: structure, function, and physiological roles. Pharmacol Rev.

[14] Khan S, Sur S, Newcomb CJ, Appelt EA, Stupp SI (2012). Self-assembling glucagon-like peptide 1-mimetic peptide amphiphiles for enhanced activity and proliferation of insulin-secreting cells. Acta Biomater.

[15] Baker AE, Sague S, Grygiel TLR, Schmidt A, Rogers A, Jiang H, Kruszynski M, Nesspor T (2012). The dimerization of glucagon-like peptide-2 MIMETIBODY is linked to leucine-17 in the glucagon-like peptide-2 region. J Mol Recognition.

[16] Merkler DJ. C-terminal amidated peptides: production by the in vitro enzymatic amidation of glycine-extended peptides and the importance of the amide to bioactivity. Enzyme Microb Technol. 1994, 16:450**–**456. 10.1016/0141-0229(94)90014-0 7764886

[17] Tamburini PP, Jones BN, Consalvo AP, Young SD, Lovato SJ, Gilligan JP, Wennogle LP, Erion M, Jeng AY (1988). Structure-activity relationships for glycine-extended peptides and the alpha-amidating enzyme derived from medullary thyroid CA-77 cells. Arch Biochem Biophys.

[18] Wulff BS, Catipovic B, Okamoto H, Gether U, Schwartz TW, Johansen TE. Efficient amidation of C-peptide deleted NPY precursors by non-endocrine cells is affected by the presence of Lys-Arg at the C-terminus. Mol Cell Endocrinol. 1993, 91:135**–**141. 10.1016/0303-7207(93)90265-l 8472845

[19] Ciccotosto GD, Schiller MR, Eipper BA, Mains RE (1999). Induction of integral membrane PAM expression in AtT-20 cells alters the storage and trafficking of POMC and PC1. J Cell Biol.

[20] Husten EJ, Tausk FA, Keutmann HT, Eipper BA (1993). Use of endoproteases to identify catalytic domains, linker regions, and functional interactions in soluble peptidylglycine alpha-amidating monooxygenase. J Biol Chem.

[21] El Meskini R, Culotta VC, Mains RE, Eipper BA (2003). Supplying copper to the cuproenzyme peptidylglycine α-amidating monooxygenase. J Biol Chem.

[22] Handa S, Spradling TJ, Dempsey DR, Merkler DJ (2012). Production of the catalytic core of human peptidylglycine α-hydroxylating monooxygenase (hPHMcc) in *Escherichia coli*. Prot Expr Purif.

[23] Siebert X, Eipper BA, Mains RE, Prigge ST, Blackburn NJ, Amzel LM (2005). The catalytic copper of peptidylglycine α-hydroxylating monooxygenase also plays a critical structural role. Biophysical J.

[24] Chufan EE, De M, Eipper BA, Mains RE, Amzel LM (2010). Amidation of bioactive peptides: the structure of the lyase domain of the amidating enzyme. Structure.

[25] Eipper BA, Stoffers DA, Mains RE (1992). The biosynthesis of neuropeptides: peptide alpha-amidation. Annu Rev Neurosci.

[26] Guest PC, Bailyes EM, Rutherford NG, Hutton JC (1991). Insulin secretory granule biogenesis. Biochem J.

[27] Farquhar MG, Palade GE. The Golgi Apparatus (Complex) - (1954–1981) - from Artifact to Center Stage. J Cell Biol. 1981, 91:77–103. 10.1083/jcb.91.3.77s PMC2112780 7033246

[28] Gerald C, Walker MW, Criscione L, Gustafson EL, Batzl-Hartmann C, Smith KE, Vaysse P, Durkin MM, Laz TM, Weinshank RL (1996). A receptor subtype involved in Neuropeptide-Y-induced food intake. Nature.

[29] Prigge ST, Kolhekar AS, Eipper BA, Mains RE, Amzel LM (1997). Amidation of bioactive peptides: the structure of peptidylglycine α-hydroxylating monooxygenase. Science.

[30] Milgram SL, Johnson RC, Mains RE (1992). Expression of individual forms of peptidylglycine alpha-amidating monooxygenase in AtT-20 cells: endoproteolytic processing and routing to secretory granules. J Cell Biol.

[31] Rajagopal C, Stone KL, Francone VP, Mains RE, Eipper BA (2009). Secretory Granule to the Nucleus. J Biol Chem.

